# Effects of DMT on mental health outcomes in healthy volunteers

**DOI:** 10.1038/s41598-024-53363-y

**Published:** 2024-02-07

**Authors:** Christopher Timmermann, Richard J. Zeifman, David Erritzoe, David J. Nutt, Robin L. Carhart-Harris

**Affiliations:** 1 Centre for Psychedelic Research, Department of Brain Sciences, Faculty of Medicine, Imperial College London, London, UK; 2 NYU Langone Center for Psychedelic Medicine, NYU Grosssman School of Medicine, New York, USA; 3 Centre for Psychiatry, Division of Brain Sciences, Faculty of Medicine, Imperial College, London, UK; 4 Psychedelics Division, Neuroscape, Department of Neurology, University of California, San Francisco, USA

**Keywords:** Medical research, Psychology

## Abstract

Psilocybin, a serotonergic psychedelic, is being increasingly researched in clinical studies for the treatment of psychiatric disorders. The relatively lengthy duration of oral psilocybin’s acute effects (4–6 h) may have pragmatic and cost-effectiveness limitations. Here, we explored the effects of intravenous (IV) N,N-Dimethyltryptamine (DMT), a closely related, but faster-acting psychedelic intervention, on mental health outcomes in healthy volunteers. Data is reported from two separate analyses: (1) A comparison of mental health-related variables 1 week after 7, 14, 18, and 20 mg of IV DMT versus IV saline placebo (n = 13) and, (2) A prospective dataset assessing effects before versus 2 weeks after 20 mg of IV DMT (n = 17). Mental health outcomes included measures of depression severity (QIDS-SR16), trait anxiety (STAI-T), Neuroticism (NEO-FFI), wellbeing (WHO-5), meaning in life (MLQ), optimism (LOT-R), and gratitude (GQ-6). In both the prospective and placebo-controlled datasets, significant improvements in scores of depression were found 1–2 weeks after DMT administration. Significant reductions in trait Neuroticism were only found for the placebo-controlled sample. Finally, changes in depression and trait anxiety correlated with acute peak experiences (assessed via ‘Oceanic Boundlessness’). While the use of two separate cohorts in pooled analysis limits the generalizability of these correlational findings, these results suggest that DMT may reduce depressive symptomatology by inducing peak experiences. The short half-life of IV DMT and its potential for flexible dosing via controlled infusions makes it an appealing candidate for psychedelic medicine. Further research in clinical samples is needed to corroborate the therapeutic potential of DMT.

## Introduction

Mental illness is a major global public health problem, with affective disorders (e.g., anxiety and depression) being among the leading causes of disability^1^. Given the limitations surrounding current treatments, there is a need for exploring novel interventions^2^. Accordingly, it is noteworthy that classic psychedelic (i.e., serotonin 2A receptor [5-HT2AR] agonist) therapy has been found to improve a range of mental health outcomes (e.g., for a review, see Andersen et al.^3^).

To date, research on the therapeutic utility of classic psychedelics has primarily focused on psilocybin, lysergic acid diethylamide (LSD), and ayahuasca (a brew that contains the β-carboline alkaloids harmine, harmaline and tetrahydroharmine, as well as N,N-Dimethyltryptamine—DMT^4^). Psilocybin administration with psychological support has been found to reduce depression and anxiety symptoms in open-label and controlled trials, with effects lasting up to six months in some studies^5–11^. Similarly, in a placebo-controlled trial, LSD with psychological support was recently found to be effective in reducing symptoms of depression and anxiety^12^. Finally, the DMT-containing brew ayahuasca was also found to reduce symptoms of depression^13^. Findings in controlled studies are supported by evidence from naturalistic research involving large and diverse populations. Together, evidence strongly suggests that, used with care, classic psychedelics can have a positive impact on factors that traverse the mental health spectrum, by elevating psychological well-being^14–18^, gratitude^19–21^, optimism^10,21–23^, mindfulness capacities^24–26^, and meaning in life^10,21^. These studies have primarily focused on psilocybin, LSD and 5-MeO-DMT (a compound related to DMT, which induces somewhat different subjective effects)^27^. Within a very small sample (n = 6), one recent study found significant decreases in depression 1 day after DMT administration^28^. While larger (but still small) samples have been used in studies employing DMT as a component of the ayahuasca brew^13,29^, the latter also contains other psychoactive ingredients (including monoamine oxidase inhibitors such as harmine and harmaline) that may also contribute to the therapeutic potential of the brew. Considering these limitations of small sample sizes and drug confounds, the therapeutic utility of DMT monotherapy has not been adequately assessed.

DMT has a somewhat distinct profile from other classic psychedelics^30,31^. At standard doses, DMT produces an especially intense acute experience that includes vivid visual imagery. These include subjectively-felt encounters with ‘alternate’ dimensions and beings^32,33^, intense proprioceptive or somatosensory effects, and near-death-like experiences^34^. Perhaps the most important distinction between DMT and the other classic psychedelics currently being studied (e.g. psilocybin, LSD) is its short acute duration when injected or inhaled^35^, and apparent lack of tachyphylaxis or desensitization with use^36^. The acute effects of orally ingested classic psychedelics generally last from 4 to 10 h^37^ but intravenous (IV) and smoked DMT has an acute duration of approximately 20 min^35^. Furthermore, DMT’s apparent lack of tachyphylaxis allows for the possibility of flexibly adapting its administration to variable durations^38^. Given concerns regarding the cost and large-scale accessibility of lengthy and amply staffed psychedelic therapy sessions (e.g., traditionally involving two therapists for 7–10 h), compounds with briefer durations, like IV DMT, may be worth exploring^25,27,39^. This may be especially relevant for patients who experience a recurrence of their clinical symptoms following psilocybin therapy (see^5,8^ for examples) and may benefit from shorter (e.g., pro re nata) interventions as treatment follow-ups.

The present report, consisting of two separate studies, aimed to examine the impact of IV DMT on assessments of: (1) negative psychological factors, including depression severity, trait anxiety, and trait Neuroticism (commonly implicated in psychiatric conditions) in psychedelic-experienced healthy volunteers; and (2) positive psychological factors associated with mental health (i.e., well-being, optimism, nature-relatedness, gratitude, and meaning in life). Here, we present results from a placebo-controlled sample in which we assessed the impact of DMT on mental health outcomes, complemented by results from a prospective sample. While the use of separate cohorts represents strengths in replicating findings when analysed separately, the generalizability of findings may be limited when samples are pooled for analyses. Here we perform both replication and pooled analyses. Considering the early stages of DMT research in human volunteers, this study recruited only psychedelic-experienced healthy volunteers to ensure psychological safety, while providing preliminary evidence for the potential benefits of DMT on mental health.

## Results

### Improvements in depression symptoms post-DMT administration

For the samples presented here, DMT was administered over two studies. The context of use was neuroimaging studies. These studies resulted in a placebo-controlled sample, and a prospective sample (see “Methods”). For the placebo-controlled sample, mental health-related variables were measured at baseline, 1 week after placebo and 1 week after DMT (the order of administration was fixed with placebo being administered before and DMT, after). Differences in depression (QIDS-SR16) and trait anxiety (STAI-T) were assessed 1 week after placebo vs 1 week after baseline, as well as 1 week after DMT vs 1 week after placebo administration (DMT and placebo were administered to the same participants). For the prospective sample, differences in these outcomes were compared by contrasting DMT (taken 2 weeks after administration) against baseline. This baseline comparison was chosen due to lack of a plausible placebo condition in the second study; see “Methods”.

**Figure 1:**
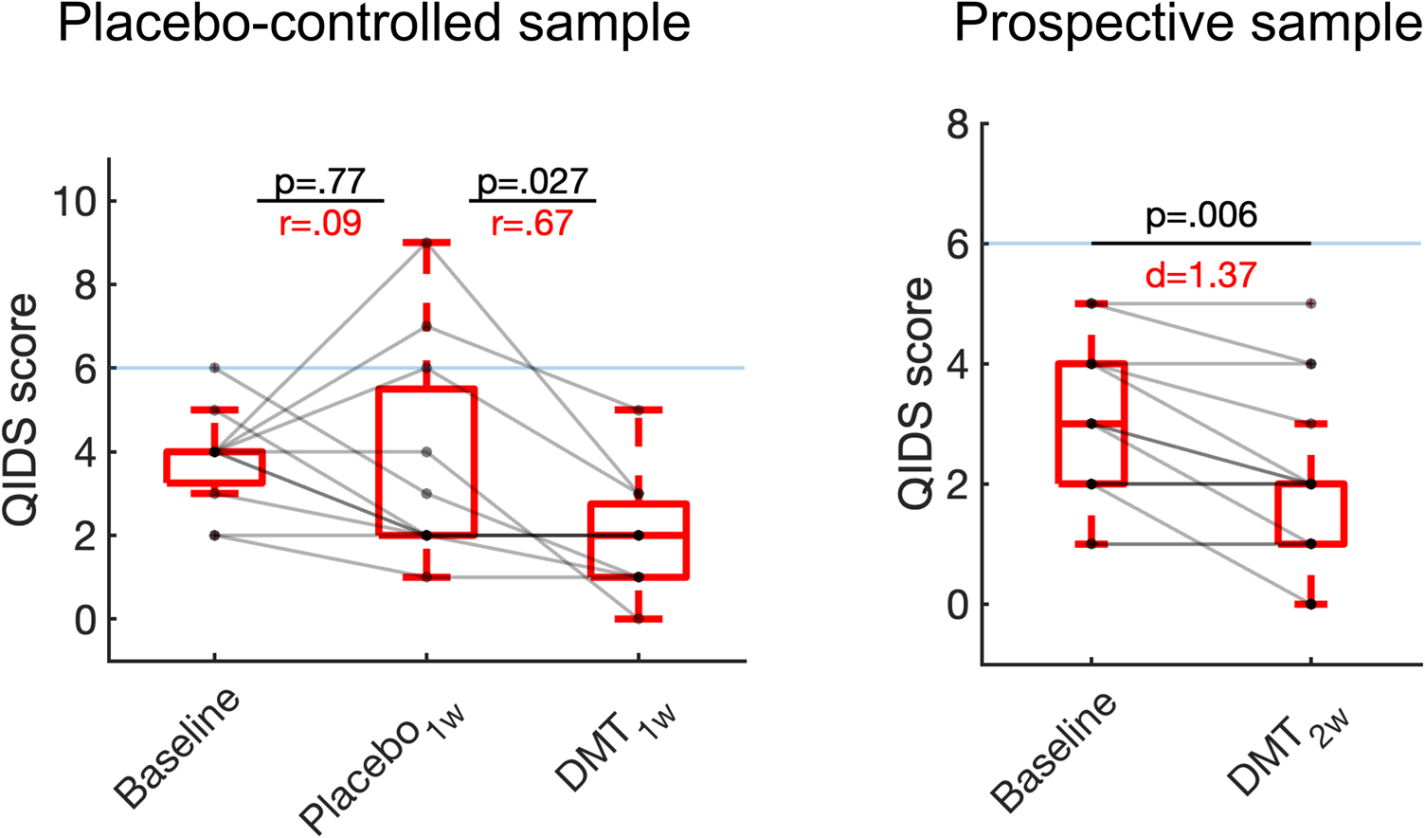
Effects of DMT on depression severity. Boxplots displaying significant reductions for depression symptomatology (QIDS-SR16; the horizontal blue line marks threshold for mild depression) after DMT for placebo-controlled (left) and prospective (right) samples. No significant differences were observed in the comparison of placebo and baseline for the placebo-controlled sample. P-values (two-tailed) and effect sizes are displayed.

Consistent with our main hypothesis, significant reductions in depression were observed 1 week following DMT administration relative to 1 week after placebo (z[10] = − 2.06, p = 0.027 two-tailed, *r* = 0.67)—whereas no significant differences were observed in the comparison between 1 week after placebo and baseline (z[10] = − 0.29, p = 0.77 two-tailed, *r* = 0.09)—in the placebo-controlled sample. Within the prospective sample we also observed significant reductions in depression 2 weeks after DMT compared with baseline (t[13] = − 3.24 p = 0.0065, *d* = 1.37). While no significant reductions in trait anxiety were observed in both the placebo-controlled (DMT vs placebo t[12] = 1.61, p = 0.13 two-tailed, *d* = 0.45) or prospective samples (DMT vs baseline t[16] = − 1.58, p = 0.13 two-tailed, *d* = 0.55), medium effect sizes were found. No significant differences were found in trait anxiety for placebo versus baseline for the placebo-controlled sample (t[12] = − 0.48, p = 0.64 two-tailed, *d* = 0.13) (Fig. 1).

Consistent with improvements in depressive symptoms, significant reductions were found for the personality domain Neuroticism following DMT administration compared with placebo in the placebo-controlled sample (t[12] = − 2.60, p = 0.02 two-tailed, d = 0.72), however, no significant differences were found for the comparison between 2-weeks post DMT vs baseline in the prospective sample (t[16] = − 1.48, p = 0.16 two-tailed, d = 0.57). No significant differences were found in the comparison between placebo and baseline for the placebo-controlled sample (t[12] = − 0.67, p = 0.51 two-tailed, d = 0.19).

### Association between acute ‘Oceanic Boundlessness’ and improvements in depression/anxiety

**Figure 2:**
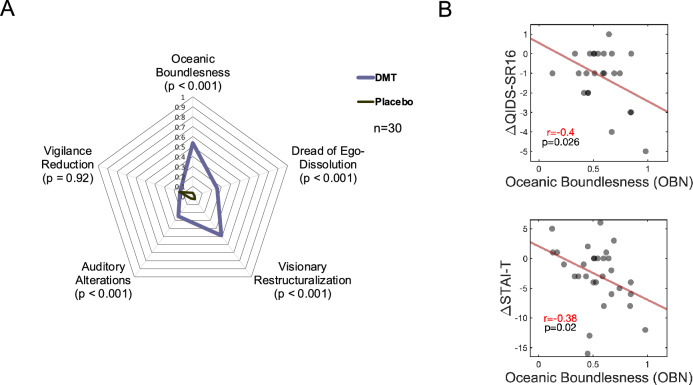
Relationship between changes in depression severity / trait anxiety and acute effects of DMT. (**A**) Radar plot displaying the acute effects of DMT vs placebo. (**B**) Correlations between DMT-induced scores in the Oceanic Boundlessness factor (OBN) and changes in depressive severity (QIDS-SR16) and trait anxiety (STAI-T). Pearson correlation r and p values are displayed, one-tailed.

Data were pooled from both studies to study the association between acute experiences induced by DMT and depression/anxiety. Compared with placebo, DMT was associated with higher scores of *Oceanic Boundlessness (OBN)*, *Dread of Ego-Dissolution*, *Visual Restructuralization*, and *Auditory Alterations* (all p < 0.001, two-tailed), however, no significant differences were found for *Vigilance Reduction* (Fig. 2A). Following Roseman et al.^40^ and Uthaug et al.^25^, we performed hypothesis-driven correlation analyses to determine the relationship between DMT-*induced* OBN versus changes in depression severity and trait anxiety in a pooled sample involving participants from both studies. A significant, inverse association was found between DMT-induced OBN and changes in depression severity (r = − 0.40, p = 0.026, one-tailed), and trait anxiety (r = − 0.38, p = 0.020, one-tailed), assessed as 1–2 weeks post-DMT minus baseline scores of QIDS-SR16 and STAI-T, respectively. One-tailed tests were used as clear hypothesis supported these analyses (Fig. 2B).

### Effects of DMT on positive psychological factors: wellbeing, nature-relatedness, gratitude, optimism, and meaning in life

While increases in nature-relatedness and gratitude were identified in the comparison of DMT against the baseline, these did not survive correction for multiple comparisons. No significant changes were seen for other positive psychological factors tested. See Table 1 for the results of all comparisons.

**Table 1:** Statistical comparisons for secondary outcomes.

Measure	Placebo-controlled sample (baseline vs placebo)		Placebo-controlled sample (placebo vs DMT)		Prospective sample (baseline vs DMT)	
	Statistic	Effect size	Statistic	Effect size	Statistic	Effect size
Wellbeing (WHO-5)	t = 0.92	*d* = 0.26	z = 1.87	*r* = 0.52	t = 0.27	*d* = 0.34
Nature-relatedness (NR-5)	t = 1.2	*d* = 0.36	t = 0.77	*d* = 0.44	z = 2.14	*r* = 0.52
Gratitude (GQ-6)	t = 0.09	*d* = 0.024	t = 1.51	*d* = 0.42	t = 2.82*	*d* = 1.23
Optimism (LOT-R)	t = 2.05	*d* = 0.57	t = 0.37	*d* = 0.10	t = 1.76*	*d* = 0.59
Meaning in life (MLQ)	t = 0.17	*d* = 0.14	t = − 0.04	*d* = − 0.01	t = 0.52	*d* = 0.19

The table shows statistical comparisons between 1 week post-DMT vs 1 week post-placebo (placebo-controlled data) and between 2 weeks post-DMT vs baseline. *p < 0.05 two-tailed (uncorrected). No significant comparisons survived Bonferroni correction for multiple comparisons.

## Discussion

Here we report mental health outcomes in relation to IV DMT in two separate studies. Reductions in depression severity were observed in both a small placebo-controlled study, and a larger sample from just the DMT arm. These results were supplemented by reductions in trait Neuroticism in the placebo-controlled sample. Consistent with previous psychedelic research implicating a causal role for experiential factors in relation to mental health changes, a significant relationship was found between acute experiences of ‘Oceanic Boundlessness’ induced by DMT and post-DMT improvements in depression and anxiety. Together, these results tentatively support further research into the therapeutic potential of DMT in psychiatry.

Depression symptomatology was the main outcome assessed in a number of recent clinical trials involving psychedelics, and they have consistently been found to be reduced after psychedelic therapy in psychiatric patient populations^6–8,10,11,13,41,42^ and in healthy volunteers in retrospective surveys^25,27,43^. Our current findings are consistent with reductions in depression symptoms found in previous studies, as well as others that have reported reductions in the Neuroticism personality domain following the administration of psychedelics to individuals with depression^44^. Interestingly, a recent study showed evidence for reduced depression in six participants with major depression 1 day after 21 mg/70 kg of DMT administration^28^, consistent with preclinical behavioural models of depression^45^.

Tempering enthusiasm, we failed to replicate decreases in Neuroticism in the prospective sample, and we did not find increases in positive psychological factors such as well-being, optimism, gratitude, nature-relatedness, and meaning in life after DMT, which is contrary to the findings of several controlled and non-controlled healthy volunteers^14–17,21,25,46–48^ and clinical studies^10,21^ involving psychedelics. Speculatively, one could argue that a more therapeutically supportive context (e.g., not being inside of an MRI scanner with an EEG cap for a neuroscience experiment) or a longer experience (such as the one enabled by longer infusions of DMT) may be necessary for larger improvements in these measures. Recent evidence suggests that the effects of DMT can be extended by at least 30 min^38,49^ (and thus, the efficacy of therapeutic action may also be elevated) while still inducing effects significantly shorter than those induced by psilocybin and LSD. There is also the possibility that the effects of DMT on mental health outcomes have been limited by ceiling effects from an already healthy population.

Finally, we found that decreases in depression and anxiety were significantly associated with the quality of the acute experiences induced by DMT, as measured by the 5D-ASC subscale *Oceanic Boundlessness*. This finding is consistent with a large number of studies that have found positive relationships between acute ‘peak’, ‘mystical’ or emotional experiences and improvements in mental health outcomes with psychedelics^13,15,16,25,40,50–53^. Future work is needed to assess whether other variables that have been shown to contribute to improved mental health outcomes with psychedelics, such as insight^54,55^, emotional breakthrough^56^, and psychological flexibility^57,58^, play a similar role with DMT.

As with other psychedelics, psychological mechanisms underlying the therapeutic potential of DMT are complemented by neurobiological mechanisms of action. As with other psychedelics, DMT is primarily associated with stimulation of 5-HT2A receptors^59^, while most conventional antidepressants increase serotonin receptor agonism non-selectively. It has been proposed that 5-HT2AR agonist psychedelics can be effective in the treatment of depression (and other psychiatric disorders) via their ability to relax and (with psychological support) revise entrenched pathological habits of mind and/or behaviour^60,61^. In contrast, conventional antidepressants have been proposed to promote a type of ‘passive coping’, incubating against stress (e.g., via stimulation of inhibitory post-synaptic 5-HT1A receptors in stress circuitry)^60,62^. Harnessing increased cortical plasticity^63^ to aid the revision of cognitive and behavioural habits, could lead to more enduring benefits with psychedelic therapy compared with conventional antidepressants.

It is important to note some limitations of the present studies. Firstly, in the placebo-controlled sample, different doses were used, which may have generated variability in the outcomes we reported. Secondly, the studies differed in methodology and timing of assessment of mental health outcomes (see “Methods”), which may have increased this variability. This becomes particularly relevant when the datasets are pooled for analysis examining the predictive relationship of Oceanic Boundlessness scores and mood symptomatology, and thus limits the generalizability of this correlational finding. Thirdly, data from an EEG-fMRI study consisting of a prospective dataset and this study design did not allow for an assessment of the effect of a placebo session on mental health outcomes, as all study volunteers received both DMT and placebo on the same study visit and no follow-ups for a placebo condition were possible. We were therefore limited to (prospective) baseline (pre DMT) versus post DMT contrasts on these analyses. Despite this limitation, the findings are largely consistent with the placebo-controlled findings we also report, as well as those from other placebo-controlled studies with DMT^28^ and ayahuasca^13^. Moreover, our studies were primarily neuroscientific rather than therapeutic—and previous work has shown that having a therapeutic intent and receiving the psychedelic in a therapeutic context positively modulates relevant mental health outcomes^64^, whereas neuroimaging environments (such as an fMRI environment in the second study) are associated with less positive responses^65^. Thus, further work is needed to assess whether DMT is equally sensitive to contextual modulation and whether the present signal can be improved upon as we suspect it can. Thus, for several reasons, the present findings may under-estimate the ‘true' magnitude of improvement possible with IV DMT.

Relatedly, it is important to note that these studies were conducted with psychedelic-experienced healthy volunteers and not individuals with a psychiatric disorder, and thus these findings may not extend to psychedelic-naïve clinical populations. To attend to this discrepancy (and prevent floor effects which would hinder statistical analysis), we removed individuals scoring the most extreme score possible on mental health outcomes from further analyses. While this may limit this sample representativeness’ for healthy populations, we believe it better informs the potential applicability of DMT for individuals with mood symptomatology.

While studies employing structurally-similar compounds are showing promising results in clinical populations^8,10,11,13,41,42^, it is important to note that there might be additional challenges with the employment of DMT for clinical purposes. These include: (1) the short-duration of action associated with DMT administration compared with other psychedelics, as it is reasonable to assume that a longer experience is required for therapeutic benefits in certain difficult-to-treat clinical populations^66^. This possibility may be implied by some recent evidence suggesting an important role for psychological insight in moderating responses to psychedelics^55^; and (2) DMT is associated with especially extravagant psychological effects (e.g. exploring ‘alternate realms’ and sensing other sentient presences or entities) that while intense, are so short-lived that insight may be compromised^32,34^.

Special consideration might be required for helping individuals ‘integrate’ the unusual psychological effects of DMT in order to remain psychologically grounded. An open-label study employing psilocybin for treatment-resistant depression found effect sizes that were 2–3 times larger compared with the effect sizes we report here using the same QIDS-16-SR measure^5^. The statistical phenomenon known as ‘regression to the mean’—i.e., that extreme values gravitate towards the average with repeat measures—may partly account for this, as well as ‘floor effects’ when baseline symptom severity scores are low-to-negligible in healthy populations. For these reasons, we suspect that larger effect sizes may be observed if a population with more severe anxious/depressive symptoms was recruited for a DMT trial.

In conclusion, the present study tentatively suggests that intravenous DMT may have some utility in improving mild symptoms of depression in already healthy populations. Our findings are generally consistent with those from recent clinical trials involving other psychedelics, as well as healthy volunteer studies. Rapid-acting, short half-life psychedelics are appealing candidates for further research due to their potentially superior cost-effectiveness, as well as their potential for flexible dosing. Further research is required to examine whether short-acting psychedelic interventions, such as DMT, have consistent safety and efficacy profiles.

## Methods

### Study design and participants

Here we present data from 30 healthy volunteers who received IV DMT fumarate in two separate studies (see Table 2 for participants’ demographics). Participants from both studies were recruited via word-of-mouth and attended a screening visit consisting of routine physical tests, plus a psychiatric interview and medical examination. The main exclusion criteria were: having no previous experience with a psychedelic drug, current or previously diagnosed psychiatric illness, immediate family history of psychotic disorder, excessive use of alcohol (> 40 weekly units), blood or needle phobia, or a significant medical condition rendering volunteers unsuitable for participation (e.g., diabetes, heart condition). The assessment of no current or previously held psychiatric illness was determined via a thorough psychiatric interview, which included the MINI-mental, rather than via the administration of the QIDS scale. All participants provided written informed consent for participation in the study. This study was approved by the National Research Ethics (NRES) Committee London—Brent and the Health Research Authority and was conducted under the guidelines of the revised Declaration of Helsinski (2000), the International Committee on Harmonisation Good Clinical Practices guidelines, and the National Health Service Research Governance Framework. Imperial College London sponsored the research, which was conducted under a Home Office license for research with Schedule 1 drugs.

**Table 2:** Demographic characteristics of participants included in the analyses.

Sample	Average age in years (standard deviation)	Gender	Ethnic origin	Nationality	Marital status	Employment	Education	Years in education (standard deviation
Placebo-controlled	34.76 (9.44)	46.2% female, 53.8% male	92.7% White, 7.7% Turkish	69.2% British, 7.7% Serbian, 7.7% Danish, 7.7% Chilean, 7.7% Turkish	61.5% single, 30.8% married, 7.7% did not answer	69.2% skilled, 15.4% student, 7.7% unskilled, 7.7% other	69.2% degree, 30.8% other	23.58 (10.95)
Prospective	33.12 (7.57)	41.18% female, 58.82% male	64.7% White, 11.8% Asian, 11.8% not answered, 5.9% Black, 5.9% other	47.1% British, 11.8% Hungarian, 11.8% German, 5.9% Dutch, 5.9% Chilean, 5.9% Russian, 5.9% American, 5.9% French	64.7% single, 23.5% married, 5.9% divorced, 5.9% not answered	70.6% skilled, 29.4% student	100% degree	18.82 (5.22)

### Placebo-controlled sample

The placebo-controlled sample corresponds to data from a placebo-controlled, single-blind experiment in which DMT and placebo were administered to 13 healthy volunteers (6 female, 7 male, mean age = 34.76, SD = 9.44) in a fixed order (this allowed individuals to acclimatize to the research environment in this initial study). As part of a dose-finding protocol, four different doses of DMT were administered. Three volunteers received 7 mg, four received 14 mg, one received 18 mg, and five received 20 mg DMT. DMT was administered intravenously (IV) in a 2 ml sterile saline solution over 30 s (i.e., bolus injection), which was then flushed with 5 ml of saline over 15 s. Placebo (an IV administration of 2 ml of saline solution and flushed with a 5 ml of saline) was always given in the first session and DMT in the second in order to avoid carry-over effects. Sessions were separated by 7 days. During each dosing session, participants lay comfortably in supine position while electroencephalography (EEG) recordings took place. The subjective effects of DMT lasted on average 17 min. Peak effects were reached 2–3 min following injection^33^. After 20 min, participants completed questionnaires and were interviewed, with questions focused on the drug’s subjective effects. Questionnaires assessing mental health outcomes were completed electronically at baseline (one day before the placebo session), 1 week after the placebo session (range 5–7 days), and 1 week after the DMT session (range 6–9 days). Considering the sample size and strong variability of DMT levels found in plasma even at consistent doses^67^, we collapsed across doses for statistical comparisons. The main results presented here correspond to this placebo-controlled study.

### Prospective sample

The prospective sample corresponds to data from a placebo-controlled, single-blinded, EEG-fMRI study with a pseudo-balanced order. Twenty-five participants (10 female, 15 male, mean age 33.4, SD = 7.9; characteristics of the final sample included in the analyses can be found in Table 2) underwent a total of four dosing sessions on two separate days (with two dosing sessions on each day). Dosing visits were separated by 2 weeks (range = 11–14 days) and, on each dosing day, dosing sessions were separated by 4 h approximately. IV DMT was administered in a 5 ml sterile saline solution over 30 s (i.e., bolus injection), which was then flushed with 5 ml of saline over 15 s. Placebo consisted of IV administered 5 ml saline solution given over 30 s and flushed with 5 ml of saline over 15 s.

During the first visit, half of the participants received DMT in the first dosing session and placebo in the second dosing session. The order was reversed for the other 50% of participants. Considering that anxiety was elevated only mildly in the first study, we favoured a counterbalanced order in this second study to control for order effects. Sessions were separated by at least 2 h to allow participants to complete questionnaires and interviews assessing subjective effects of DMT/placebo. For the second visit, the same dosing protocol was followed but in reversed order (i.e., those who had previously received DMT first now received placebo first). Participants received DMT/Placebo while wearing an EEG cap and lying inside of a magnetic resonance imaging (MRI) scanner. Questionnaires assessing mental health outcomes were completed electronically at baseline (1 day prior to the first dosing session) and 2 weeks after the first dosing session (range 12–15 days), which always involved receipt of DMT. It is important to note that for the prospective sample participants could not answer questionnaires related to mental health with reference to the placebo session—because participants received both a dose of placebo and DMT on this first study visit (i.e., they received DMT on both of two study visits). Therefore, no placebo follow-up measurement was possible for these participants. However, they were able to provide psychometric scores corresponding to the acute experiences elicited by both DMT and placebo as shown in Fig. 2A.

Four participants dropped out from the study corresponding to the prospective sample and thus could not be asked to complete follow-up administration of questionnaires: one participant was excluded due to excessive motion in the scanner, the other dropped out due to discomfort being inside the scanner for the total duration of the experiment (28 min), and two others failed to show up (adverse reactions were not observed in these cases and unpleasant effects of DMT were not mentioned as the cause for dropping out). One participant only partially completed the study questionnaires; this data was excluded from analysis.

### Measures and outcomes

The following self-rated measures were employed: Quick Inventory for Depressive Symptomatology (QIDS-SR16^68^), the trait scale of the State-Trait Anxiety Inventory (STAI-T^69^), and the Neuroticism scale from the Neuroticism-Extraversion-Openness Five-Factor Inventory (NEO-FFI)^70^. Secondary outcomes consisted of: the World Health Organization-Five Well-Being Index (WHO-5^71^); Life Orientation Test-Revised (LOT-R; a measure of optimism^72^); Gratitude Questionnaire (GQ-6^73^), and Meaning in Life Questionnaire (MLQ^74^). The acute effects of DMT were assessed using the 5-Dimensional-Altered States of Consciousness Questionnaire (5D-ASC^75^) and only the *Oceanic Boundlessness* dimension (OBN) was used for correlational analyses. The OBN dimension, an index of peak or mystical-type experiences, has previously been found to predict changes in clinical outcomes^25,40^ and specific hypothesis were held regarding the inverse association between DMT-induced OBN and reductions in depression severity and trait anxiety (1–2 weeks post-DMT minus baseline scores of QIDS-SR16 and STAI-T, respectively), and thus no multiple comparisons corrections were performed for correlational analysis.

### Statistical analysis

Analyses examining changes in mental health outcomes were performed separately for the placebo-controlled and prospective samples. For the placebo-controlled sample, comparisons were performed contrasting 1 week post DMT vs 1 week post- placebo. For the prospective sample contrasts were made contrasting 2 weeks post-DMT scores against baseline scores. Age (p = 0.60) and gender (p = 0.66) did not differ statistically between participants of both studies (individuals who participated in both studies were excluded from the prospective sample for analysis of changes in mental health).

For normally-distributed data, paired t-tests were used to compare scores post DMT versus placebo (placebo-controlled sample analysis) and post-DMT versus baseline (prospective sample analysis). Considering that the samples consisted of healthy individuals - and following our previous work measuring outcomes in depression in a prospective non-clinical sample^76^ to avoid floor/ceiling effects - participants displaying minimum/maximum scores (corresponding to optimal mental health scores) in the placebo/baseline conditions (which always preceded the administration of DMT) were excluded from analyses to avoid floor/ceiling effects. Consequently, two participants from the placebo-controlled data, and three form the prospective data displaying a minimum score (i.e., zero) were removed for QIDS-SR16 comparisons; and two participants from both datasets displaying a maximum score (i.e., five) were removed for NR-5 comparisons. Whenever data was not normally distributed, a Wilcoxon Rank Sum test was used (Anderson–Darling goodness-of-fit test was performed to establish normality). Separate t-tests were performed for each of the subscales of the 5D-ASC, measuring acute effects of DMT versus placebo.

Based on previous evidence showing a significant impact of acute psychedelic experiences on mental health outcomes^66^, Pearson-Point correlation analyses were performed between the *Oceanic Bo*undlessness scores from the 5D-ASC^75^ and scores on the QIDS-SR16 and STAI-T pooling participants from both samples. Pooling was performed in order to increase statistical power and avoid spurious correlations which may arise from the reduced sample sizes of each study. While the pooling of participants from both studies could be questioned due to the variable doses used in the placebo-controlled study, previous studies employing such correlations with users using variable doses of psychedelics support the notion that the key variable assessed is the quality of experience—measured via scores in Oceanic Boundlessness—rather than the dose itself^15,25^, The hypothesis that can be derived from this previous work is that variable doses would induce variability of scores in this subscale and thus variable mental health outcomes, further validating the pooling of samples administered with separate doses. Nonetheless, in order to maximise the homogeneity of the doses administered, for correlational analyses we retained only the data from the prospective sample for subjects who participated in both studies, as the dosage was fixed to 20 mg only for the prospective sample.

Based on previous findings reviewed in the introduction, primary outcomes consisted of testing specific hypotheses regarding decreases in depression, anxiety, and Trait Neuroticism measured with QIDS-SR16, STAI-T, and the Neuroticism factor in the NEO-FFI post-DMT compared to placebo/baseline, and therefore no multiple comparisons correction was performed for these analyses. For all other paired t-tests corresponding to non-clinical measures (i.e., WHO-5, NR-5, GQ-6, LOT-R, MLQ), Bonferroni-correction for multiple comparisons were performed. For these analyses, significance was established at p < 0.05 (two-tailed). Effect sizes were calculated using *Cohens’ d and r* for all paired t-tests, and Wilcoxon tests, respectively. Based on previous work^25,40^, specific hypotheses were held for correlational analyses between scores in the Ocean Boundlessness scale and depression and anxiety symptomatology (determined via QIDS and the STAI-T, respectively), and thus significance was established at p < 0.05 (one-tailed) and no multiple comparisons correction was performed.

## Data Availability

The datasets used and analysed during the current study available from the corresponding author on reasonable request.
